# Hydrogen Bonds

**DOI:** 10.3390/molecules28041616

**Published:** 2023-02-08

**Authors:** Mirosław Jabłoński

**Affiliations:** Faculty of Chemistry, Nicolaus Copernicus University in Toruń, Gagarina 7, 87-100 Toruń, Poland; teojab@chem.umk.pl; Tel.: +48-56-611-4695

The Topical Collection “Hydrogen Bonds” is a continuation of the previous Special Issue “Intramolecular Hydrogen Bonding 2021” [1]. Nevertheless, there is a crucial difference: by also including intermolecular hydrogen bonds, the topic is significantly generalized. This collection consists of 12 research articles, including one review article. Once more, the collected articles address various aspects of hydrogen bonding, which are analyzed by both experimental and various theoretical methods; these articles demonstrate that hydrogen bonding remains a major topic in chemistry.

Water is a quintessential example of the occurrence of hydrogen bonding; therefore, Weinhold’s theoretical study on high-density water clusters [2] deserves special attention. The water clusters considered in this article are formed via the use of a cyclic (H${}_{2}$O)${}_{4}$ “windowpane” cluster, characterized by a quadrilateral coordination motif and then by limiting itself to the requirement of the maximal Grotthuss-type proton ordering and organization according to the Aufbau fashion. Importantly, Weinhold demonstrates the utility of the NRT-based bond order and emphasizes that the nature of hydrogen bonding can be completely explained in terms of resonance-covalency (“charge transfer”).

By applying quantum chemical topology (QCT) tools (QTAIM, IQA) and electronic delocalisation indicators (FLU and MCI), Gallegos, Barrena-Espés, Guevara-Vela, Rocha-Rinza, and Pendás detail the influence of aromaticity and antiaromaticity on the characteristics of the so-called aromaticity and antiaromaticity-modulated hydrogen bonds (AMHB) [3]. Significant differences are observed, depending on whether a proton donor or proton acceptor atom is incorporated into the monomer ring. The results that the authors obtain show that aromaticity and antiaromaticity may be considered on a common scale.

Based on data from both the Protein Data Bank and theoretical calculations, Pietruś, Kafel, Bojarski, and Kurczab detail the results of their research on the abundance, structure, and strength of the hydrogen bond with the fluorine atom as an acceptor [4]. The authors confirm that fluorine is a weak proton acceptor and thus forms weak hydrogen bonds; these are rather forced by the presence of stronger ligand–receptor interactions. For this reason, X-H⋯F hydrogen bonds are, typically, considerably more bent (120${}^{\circ }$–150${}^{\circ }$) than standard hydrogen bonds.

In a review article [5], Hansen shows that the influence of isotopes on NMR chemical shifts is an important tool in chemistry; isotopes can help describe hydrogen bonds, determine structural parameters, and define dimers, trimers, etc. It is particularly constructive to analyze the impact of isotopic effects upon chemical shifts in the study of common tautomeric systems. The fact that two-bond deuterium isotope effects (TBDIE) may be employed in order to estimate the energy of intramolecular hydrogen bonds is also accentuated.

The advantages of NMR spectroscopy are also exhibited in an insightful and original article by Tupikina, Sigalov, and Tolstoy [6]. Namely, the authors propose a method for determining the geometry of two coupled hydrogen bonds by defining a pair of NMR chemical shifts for various atoms. This method is based on the determination of two-dimensional maps, which present the dependence of two chemical shifts upon the position of hydrogen atoms in the coupled hydrogen bonds, and then the determination of the intersection point of two isolines. It is especially recommended that the chemical shifts of the carbons and protons in the CH groups of the linking system are used. While this method may be promising, its possible limitations are also identified.

Jabłoński presents the results of the first systematic theoretical study of the hydrogen bond between the carbene carbon atom of the commonly used imidazol-2-ylidene (I) derivatives (IMe${}_{2}$, I${}^{i}$Pr${}_{2}$, I${}^{t}$Bu${}_{2}$, IPh${}_{2}$, IMes${}_{2}$, IDipp${}_{2}$, IAd${}_{2}$) and the fundamental proton donors (HF, HCN, H${}_{2}$O, MeOH, NH${}_{3}$) [7]. The author found that, for a given carbene, the dissociation energy values of the IR${}_{2}\cdots$HD dimers increase in the following order: NH${}_{3}$< H${}_{2}$O < HCN ≤ MeOH ≪ HF; in addition, for a given HD proton donor, IDipp${}_{2}$ forms the strongest dimers. The roles of the various accompanying secondary interactions are also analyzed.

Wysokiński, Zierkiewicz, Michalczyk, Maris, and Scheiner show that the presence of the hydrogen bonds of the N-H⋯Cl type, formed between the counterion (e.g., [NH${}_{3}$-(CH${}_{2}$)${}_{4}$-NH${}_{3}$]${}^{2+}$) and the two anions [PdCl${}_{4}$]${}^{2-}$, facilitates a relatively short contact between these anions in the crystal [8]. Thus, these hydrogen bonds play the role of glue binding both anions.

Applying theoretical methods (cSAR, QTAIM, NCI), Wieczorkiewicz, Krygowski, and Szatylowicz investigate the influence of the substituent (-NH${}_{2}$ or -NO${}_{2}$ group in positions 5 and 6) and solvent (PCM model; 1 ≤ε<109) on the electronic structure and the presence of intramolecular hydrogen bonds in tautomeric forms of uracil [9]. Neither substitution nor solvent has been shown to affect tautomeric preferences significantly.

Szymański and Majerz introduce a theoretical study on the spatial structure and intramolecular hydrogen bonds of fagopyrins, which are characterized by a double-anthrone moiety and are natural photosensitizers of the *Fagopyrum* species [10]. Although these systems are characterized by the presence of a strong double hydrogen bridge O-H⋯O(=C)⋯H-O in the “peri” region, the presence of N-heterocyclic piperidine and pyrrolidine rings in these systems affects the possibility of breaking these bridges due to the formation of competitive hydrogen bonds O-H⋯N.

An essential area of manifestation of the importance of intermolecular interactions, especially hydrogen bonds, are inclusion complexes based on the guest–host interaction. Masoumifeshani, Chojecki, Rutkowska-Zbik, and Korona use energy partitioning approaches (SAPT, F-SAPT, SSMF3 (i.e., the authors’ modification of SMF)) to systematically characterize interactions in the complexes between amino acids as the potential guests and three calix[6]arene and hexa-*p-tert*-butylcalix[6]arene conformers as the hosts [11]. One of the many interesting findings of this study is that the most stable pinched-cone conformer is the one least prone to interact with amino acids. Methodologically, the SSMF3 procedure was revealed to be suited to reproducing the interaction energy of the complexes, particularly in regard to its dispersion component.

By applying Car–Parrinello and Path Integral molecular dynamics, Wojtkowiak and Jezierska investigate (also after taking into account nuclear quantum effects, NQEs) the dynamic nature of intermolecular hydrogen bonds in crystalline and gaseous phases of 2,6-difluorobenzamide, 5-hydroxyquinoline, and 4-hydroxybenzoic acid [12]. The authors reveal that the inclusion of NQEs engenders a reduction in the donor–acceptor distance and an increase in proton delocalization in hydrogen bridges (except for C-H⋯O in the first system).

Podjed and Modec, by performing in-depth solid-state studies, characterize polymorphisms in the salts of three amino alcohols (3-amino-1-propanol, 2-amino-1-butanol, and 2-amino-2-methyl-1-propanol) with quinaldinic acid (i.e., quinoline-2-carboxylic acid) [13]. All of the investigated structures contain the NH${}_{3}^{+}\cdots^{-}$OOC heterosynthon. Nevertheless, individual polymorphic forms also vary in their motifs of hydrogen bonds and π⋯π stacking interactions.
